# Effects of Chilling Treatment on Baicalin, Baicalein, and Wogonin Biosynthesis in *Scutellaria baicalensis* Plantlets

**DOI:** 10.3390/plants11212958

**Published:** 2022-11-02

**Authors:** Hyeon Ji Yeo, Chang Ha Park, Jae Kwang Kim, Ramaraj Sathasivam, Jae Cheol Jeong, Cha Young Kim, Sang Un Park

**Affiliations:** 1 Biological Resource Center, Korea Research Institute of Bioscience and Biotechnology (KRIBB), 181 Ipsin-gil, Jeongeup 56212, Korea; 2 Department of Biological Sciences, Keimyung University, Dalgubeol-daero 1095, Dalseo-gu, Daegu 42601, Korea; 3 Division of Life Sciences and Convergence Research Center for Insect Vectors, College of Life Sciences and Bioengineering, Incheon National University, Yeonsu-gu, Incheon 22012, Korea; 4 Department of Crop Science, Chungnam National University, 99 Daehak-ro, Yuseong-gu, Daejeon 34134, Korea; 5 Department of Smart Agriculture Systems, Chungnam National University, 99 Daehak-ro, Yuseong-gu, Daejeon 34134, Korea

**Keywords:** *Scutellaria baicalensis*, chilling treatment, flavones, hydrophilic metabolites

## Abstract

When plants are exposed to stressful conditions, they modulate their nutrient balance by regulating their primary and secondary metabolisms to adapt. In this study, changes in primary and secondary metabolites elicited by chilling stress treatment and the effects of treatment duration were examined in roots of *Scutellaria baicalensis* (*S. baicalensis*) plantlets. The concentrations of most sugars (maltose, glucose, sucrose, and fructose) and of several amino acids (proline and GABA), which are crucial regarding plant defense mechanisms, increased with increasing duration of chilling stress. Furthermore, salicylic acid levels increased after two-day chilling treatments, which may enhance plant tolerance to cold temperatures. The concentrations of flavones (baicalin, baicalein, and wogonin) increased during chilling stress, and those of phenolic acids (ferulic acid and sinapic acid) increased after two-day chilling treatments. The concentrations of these flavones were positively correlated with sucrose levels which acted as energy sources.

## 1. Introduction

Plant reproduction and growth are affected by a variety of environmental stressors, such as high salinity, drought, high and low temperatures, and toxins [1,2]. When exposed to a stressor, plants may acquire resistance and adapt by modulating particular physiological processes and levels of certain compounds to improve their fitness [2]. In particular, low-temperature stress, as occurs during freezing (<0 °C) and/or chilling (<20 °C), may be an important stressor regarding the development and growth of plants, particularly with respect to productivity of agricultural plants. Low-temperature stress keeps plants from expressing their whole genetic potential, directly, by inhibiting metabolic processes, and indirectly, by causing oxidative and osmotic stress [3].

*Scutellaria baicalensis* Georgi, known as Huang Qin, belongs to the Lamiaceae family and is a perennial herb. It has been used as a medicine in East Asian countries, including China. In particular, the roots of *S. baicalensis* exert medicinal effects [4]. The genus *Scutellaria* contains many herbs, which have strong physiological and pharmacological benefits, such as anticancer, anti-inflammatory, cardiovascular, antioxidant, and antibacterial effects [5].

Baicalin, baicalein, and wogonin are root-specific flavones of *S. baicalensis*. Biosynthetic pathways of baicalin and wogonin have been proposed by Zhao et al. [6,7]. Briefly, chrysin, as a precursor of these two flavones, can be generated from phenylalanine through catalysis by certain enzymes (phenylalanine ammonia lyase, cinnamic acid-specific coenzyme A ligase, chalcone synthase, chalcone isomerase, and flavone synthase II-2). For wogonin biosynthesis, chrysin is initially catalyzed to norwogonin by flavone 8-hydroxylase, after which wogonin is produced from norwogonin by flavonoid O-methyltransferases. However, the catalytic mechanism of flavonoid O-methyltransferases remains unclear. Chrysin can be catalyzed to baicalein by flavone 6-hydroxylase, and baicalin can be biosynthesized through enzymatic reactions induced by β-glucuronidase or 7-O-glucuronosyltransferase [6,7].

Several studies have proven that chilling stress enhances the accumulation of secondary metabolite production. For instance, when carrot cell suspension, *Pringlea antiscorbutica* seedling, and *Triticum aestivum* were exposed to chilling stress, the level of polyamines increased [8,9,10]. Exposure of apple peel to cold stress increases the quality and quantity of the phenolic compounds [11]. Similarly, apple trees adapted to cold climatic conditions experience the highest accumulations of chlorogenic acid. In another study, maize seedlings exposed to low temperatures experienced enhanced accumulation of anthocyanins [12]. In addition, *A. thaliana* exposed to cold stress experienced enhanced accumulation of secondary metabolites, such as anthocyanins, glucosinolates, phenylpropanoids, and terpenoids [13]. It has been reported that exposure of the leaves of the seedlings of the cold-tolerant grapevine cultivar to cold stress increases the total phenolic content and antioxidant activity [14]. Moreover, the highest accumulation of total phenolic content, total flavonoid content, and 2,2-diphenyl-1-picrylhydrazyl (DPPH) activity was achieved when the *Ocimum basilicum* plant was exposed to 4 °C for 12 h [15]. Numerous studies have been reported on secondary metabolite accumulation in various plant species, due to the effect of chilling. However, few studies have examined the effects of chilling stress, and its duration, on primary and secondary metabolites in *S. baicalensis* plantlets. Thus, the objective of this study was to investigate the influence of chilling treatment, and its duration, on metabolic alterations in *S. baicalensis* plantlets, and to determine the relationship between the respective metabolites.

## 2. Results

### 2.1. HPLC Analysis of Baicalin, Baicalein, and Wogonin in Roots of S. baicalensis Plantlets after Chilling Treatment

Baicalin, baicalein, and wogonin concentrations were quantified in the roots of *S. baicalensis* plantlets grown at 4 °C for 2, 4, and 8 d and in plantlets grown at 25 °C for 0, 2, 4, and 8 d, respectively. As shown in Table 1, baicalin content did not differ significantly between treatments. By contrast, the baicalein content showed a continuous increase, and the wogonin content gradually increased and then slightly decreased in the roots of chilled *S. baicalensis* plantlets. The highest levels of baicalin occurred in the roots of control plants (83.43 ± 7.99 mg/g dry weight (dw)), followed by those in roots of plantlets grown at 4 °C for 4 d (86.08 ± 4.21 mg/g dw) and those in roots of plantlets grown at 4 °C for 8 d (78.44 ± 14.86 mg/g dw). The highest levels of baicalein occurred in the roots of plantlets grown at 4 °C for 4 d (32.54 ± 4.20 mg/g dw) and 8 d (33.63 ± 6.55 mg/g dw), and the highest level of wogonin was observed in the roots of plantlets grown at 4 °C for 4 d (4.48 ± 0.43 mg/g dw). These findings suggested that chilling affected baicalein and wogonin biosynthesis but not baicalin biosynthesis in the roots of *S. baicalensis* plantlets.

### 2.2. HPLC Analysis of Baicalin, Baicalein, and Wogonin in Shoots of S. baicalensis Plantlets after Chilling Treatment

As shown in Table 2, only baicalin and baicalein were identified in the shoots of *S. baicalensis* plantlets grown under chilling. Compared with flavone content in the roots, the levels of baicalin and baicalein were markedly higher in the roots than in the shoots. Baicalin content did not differ significantly between the shoots of *S. baicalensis* plantlets grown at 4 °C for 4 d and those grown at 25 °C for 4 d. However, baicalin content suddenly increased after eight days of chilling treatment. By contrast, baicalein content did not change significantly during the chilling treatment. Furthermore, the highest level of baicalin was obtained in the shoots of plantlets grown at 4 °C for 8 d (2.01 ± 0.09 mg/g dw), and the highest level of baicalein was observed in plantlets grown at 25 °C for 4 d (0.17 ± 0.03 mg/g dw).

### 2.3. Metabolite Profiling of Roots of S. baicalensis after Chilling Treatment

**Figure 1:**
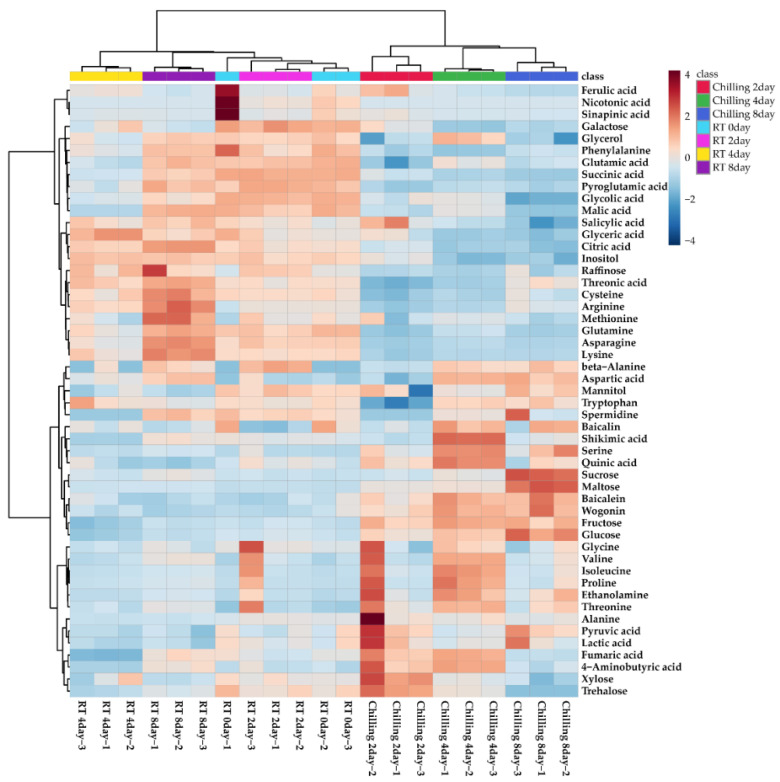
Heatmap representing differences in relative metabolite concentrations in roots of *S. baicalensis* plantlets treated with chilling. Increase and decrease in the contents of metabolites are indicated by red and blue coloration, respectively.

A total of 48 metabolites, including 2 amines, 2 phenolic acids, 3 sugar alcohols, 8 sugars, 13 organic acids, and 20 amino acids, were identified in the roots of *S. baicalensis* plantlets subjected to chilling stress, using gas chromatography time-of-flight mass spectrometry (GC-TOFMS) (Figure 1). The majority of the sugars (sucrose, maltose, fructose, and glucose) showed increasing patterns with increasing duration of chilling stress, and trehalose and xylose increased after two days of cold stress and then decreased. Furthermore, the levels of several amino acids (alanine, valine, isoleucine, proline, serine, threonine, and 4-aminobutyric acid (GABA)) increased after chilling stress. Except for these amino acids, most amino acid levels decreased or were not significantly different after chilling treatment. Regarding organic acid concentrations, pyruvic acid, shikimic acid, lactic acid, fumaric acid, and quinic acid levels increased after chilling treatment. In particular, the levels of salicylic acid, ferulic acid, and sinapic acid increased after 2 d of chilling stress and then decreased. 

**Figure 2:**
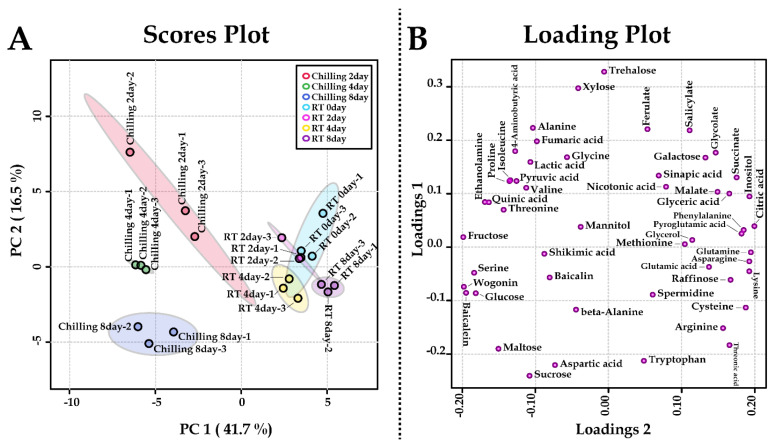
Scores (**A**) and loading (**B**) plots of the PCA model using metabolites from the roots of *S. baicalensis* plantlets treated with chilling. RT, room temperature (25 °C); ferulate, ferulic acid; succinate, succinic acid; salicylate, salicylic acid; malate, malic acid.

**Figure 3:**
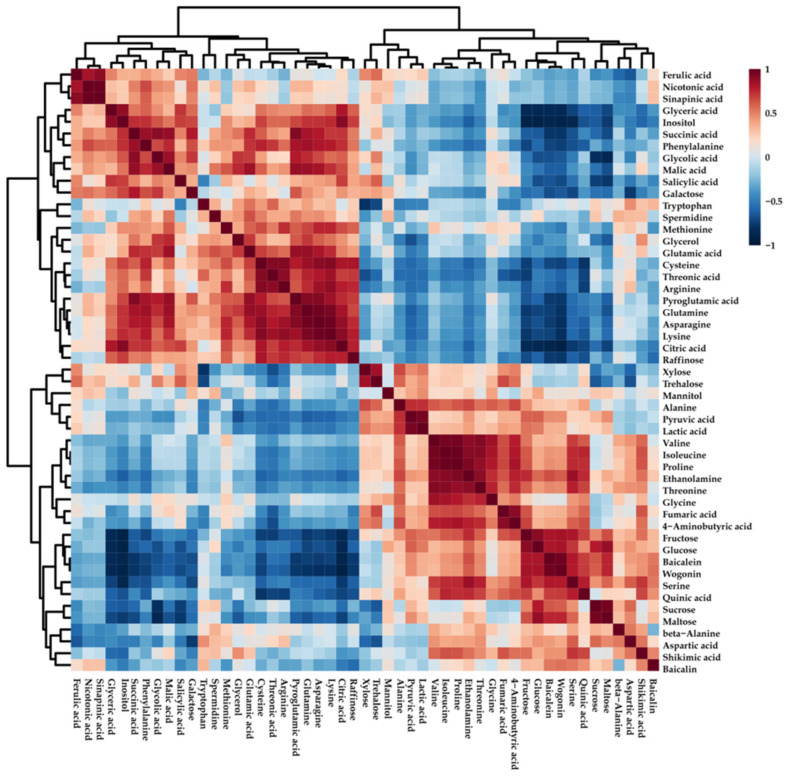
Correlation matrix of metabolites in the roots of *S. baicalensis* plantlets treated with chilling.

PCA was carried out to investigate metabolite alterations in the roots of *S. baicalensis* plantlets treated with chilling stress for different durations (Figure 2). PCA analysis revealed a clear separation between the groups representing the roots of *S. baicalensis* plantlets subjected to chilling stress at different durations and groups representing the roots of *S. baicalensis* plantlets grown at room temperature. This separation was due to the changes in sugars, amino acids, and flavones. To evaluate the relationship between the 48 metabolites detected in the roots of *S. baicalensis* plantlets subjected to chilling stress, correlation analysis was performed (Figure 3). Glutamic acid was positively correlated with its derivatives (arginine, glutamine, and pyroglutamic acid), and most sugars (sucrose, maltose, glucose, and fructose) showed strong positive correlations in the roots of *S. baicalensis* plantlets subjected to chilling stress. In particular, sucrose, which acts as an energy source for secondary metabolism, was positively correlated with baicalin, baicalein, and wogonin.

## 3. Discussion

Abiotic stressors, such as temperature, radiation, water, salinity, and nutrient stress are detrimental to plant growth and development, and plant metabolism is affected by such stressors. Secondary metabolism is involved in plant protection in response to abiotic stress [16]. It has been suggested that carbon can be mainly used for the production of defensive secondary metabolites, instead of biomass production, when plants suffer from stress [17]. The accumulation of phenolic compounds, polyamines, and amino acids in response to abiotic stresses suggests that plant metabolites function as antioxidants [16]. Cold treatment increases the content of phenolics, such as anthocyanins in maize [12] and *Arabidopsis* [18], and catechin and epicatechin in tartary buckwheat [19]. These previous observations are in line with our results, which revealed that chilling treatment increased the content of baicalin, baicalein, and wogonin in the roots of *S. baicalensis* plantlets subjected to chilling. Furthermore, this study suggests that increases in amino acids (proline and GABA) and amines (ethanolamine) are important for responses to chilling stress in the roots of *S. baicalensis*, which is supported by previous studies indicating that proline and GABA elevate plant stress tolerance [20,21] and that pretreatment with ethanolamine enhances *Helianthus annuus* seedling tolerance to saline stress [22]. In addition, the heat shock transcription factors (HSF), such as *VaHSFC1*, are involved in the protection of Amur grape plants from various abiotic stresses (cold, heat, and salinity) [23]. In *Arabidopsis*, overexpression of *VaHSFC1* improves seedling survival, reduces leaf yellowing, decreases electrolyte leakage, stimulates membrane stability, increases chlorophyll content, and enhances thermotolerance in adult plants. In addition, *TaHSF3* is over-expressed in spikes and induced by cold stress which improves the thermotolerance in bread wheat [24]. Moreover, *FGT1* has been reported to be superfluous for cold stress memory. The presence of FGT1 in metazoans suggests that it is an evolutionarily conserved protein that is involved in a wide range of responses in addition to heat shock memory [25]. From these results, it can be deduced that not only metabolites protect plants from abiotic stress, but also HSF plays an important role in plant protection from abiotic stresses. 

In the current study, chilling treatment induced an increase in baicalin, baicalein, and wogonin in the roots of *S. baicalensis* plantlets subjected to chilling, and a positive correlation was found between sucrose and these three flavones. This suggests that increased abundance of endogenous sucrose may contribute to enhanced flavone production after chilling treatment. Indeed, previous studies reported that an enhanced endogenous pool of sucrose increases the levels of phenolic compounds. For example, sucrose transporter *Arabidopsis* mutants contain more endogenous sucrose and show enhanced production of anthocyanins via upregulation of flavonoid biosynthesis genes [26]. A strong positive correlation between endogenous levels of sucrose and wogonin, baicalein, and baicalin was observed in PAP1-overexpressing hairy roots of *S. baicalensis* [27]. Furthermore, the endogenous pool of sucrose was positively correlated with phenolic acids (ferulic acid and sinapic acid) in *Lavandula pubescens* [28], with anthocyanins and carotenoids in *Morus alba* [29,30], and with galantamine in *Lycoris radiata* [31,32]. In addition, exogenous sucrose treatment enhanced the production of wogonin, baicalein, and baicalin in the hairy roots of *S. baicalensis* [33].

Salicylic acid levels increased after a two-day chilling treatment and then decreased, leading to an increase in proline production in the roots of *S. baicalensis* plantlets subjected to chilling. These findings are supported by the role of salicylic acid, which is involved in plant defenses against abiotic stresses, through the regulation of physiological processes (photosynthesis, nitrogen and proline metabolism, antioxidant defense systems, and water balance). Furthermore, salicylic acid improves tolerance to metal, salinity, osmotic, drought, heat, and cold stress [34,35].

In this study, the metabolic alteration was investigated in the roots of *S. baicalensis* plantlets during the chilling treatment. It was speculated that the metabolite changes in response to chilling might be an adaption mechanism of *S. baicalensis*. In particular, salicylic acid may enhance plant tolerance against chilling stress via regulation of proline metabolism, as seen in enhance pools of proline in the roots treated with chilling. Furthermore, the roots accumulated phenolic compounds (baicalin, baicalein, wogonin, ferulic acid, and sinapic acid) showing antioxidant capacities and GABA enhancing stress tolerance. Therefore, the results suggest that *S. baicalensis* may improve tolerance to chilling stress by modulating metabolic balance. In the future, further studies are needed to understand the functional mechanisms that link the flavonoid biosynthetic pathway genes expression and flavonoid content underlying the response to plant cold tolerance.

## 4. Materials and Methods

### 4.1. Plant Materials

In total 21 pots (11 cm × 11 cm) were filled with vermiculite and in each pot four *S. baicalensis* seeds were sown and grown for 100 days at 25 °C with an 8/16 h dark/light cycle in a normal growth chamber. Nine pots containing *S. baicalensis* plantlets were transferred to a different growth chamber for chilling treatment in which plantlets were grown at 4 °C. The remaining 12 pots were maintained in the normal growth chamber (controls). After 0, 2, 4, and 8 d, shoots and roots of plantlets were harvested from three pots of each treatment (as independent replicates) and were placed in liquid nitrogen. Subsequently, plant samples were freeze-dried (HyperCOOL HC 3055, Hanil Science Inc., Gimpo, Korea) and were ground to a fine powder for metabolite analyses.

### 4.2. HPLC Analysis of Baicalin, Baicalein, and Wogonin

Flavone concentrations (baicalein, baicalin, and wogonin) were measured in *S. baicalensis* plantlets, as previously reported [36]. Dried powder (100 mg) of shoots and roots of plants grown at 4 °C for 2, 4, and 8 d and of plants grown at 25 °C for 0, 2, 4, and 8 d were soaked in 1.5 mL aqueous methanol (80 %, *v*/*v*), followed by vigorous vortexing for 30 s. Subsequently, the mixtures were sonicated for 1 h and were centrifuged (Hanil Science Inc., Gimpo, Korea) at 10,000× *g* and 4 °C for 15 min to collect the supernatant. After syringe-filtration, filtered extracts were placed in vials. HPLC analysis (model NS-6000; Futecs, Daejeon, Korea) of baicalein, baicalin, and wogonin was performed, as reported previously [36]. Identification of baicalein, baicalin, and wogonin metabolites was performed through retention time comparison and spike tests, and they were quantified using calibration curve equations.

### 4.3. GC-TOF-MS Analysis

Hydrophilic metabolites were detected, as previously reported [37,38]. Dried powder (10 mg) of shoots and roots of *S. baicalensis* plantlets, treated at 4 °C for 2, 4, and 8 d and grown at 25 °C for 0, 2, 4, and 8 d, were mixed with 1 mL of a methanol–chloroform–water mixture (2.5:1:1, *v*/*v*/*v*) and 60 μL ribitol as an internal standard. After mixing 1200 × *g* for 1 h, the mixtures were centrifuged at 10,000× *g* for 20 min, the polar phase was evaporated in a centrifugal concentrator (CC-105, TOMY, Tokyo, Japan) for 3 h. Subsequently, derivatization was performed by adding 80 μL methoxyamine hydrochloride–pyridine (20 g L^−1^), followed by mixing at 1200× *g* and 37 °C for 2 h. After adding N-methyl-N-(trimethylsilyl)trifluoroacetamide (80 μL), the extracts were heated at 37 °C for 35 min and were transferred to vials. GC-TOFMS analysis was done by using Aglient 7890A GC (Agilent, Santa Clara, CA, USA) coupled Pegasus TOFMS 4D TOF-MS (LECO, St. Joseph, MI, USA). Analysis of hydrophilic metabolites was performed, as previously reported [37].

### 4.4. Statistical Analysis

SAS version 9.4, IBM (SAS Institute, Cary, NC, USA) was used to perform Duncan’s multiple range test [39]. MetaboAnalyst (version 5.0; http://www.metaboanalyst.ca/, accessed on 5 March 2021, was used for principal component analysis (PCA), heatmap analysis, and correlation analysis of metabolites [40].

## Figures and Tables

**Table 1:** HPLC analysis of flavones in roots of *S. baicalensis* plantlets after chilling treatment.

	Duration (Day)	Control	Chilling Treatment
Baicalin	0	63.43 ± 7.99 ab	
	2	57.71 ± 4.02 b	66.48 ± 3.27 b
	4	70.18 ± 4.36 a	86.08 ± 4.21 a
	8	65.63 ± 3.98 ab	78.44 ± 14.86 a
Baicalein	0	12.55 ± 4.96 c	
	2	9.09 ± 3.91 c	22.28 ± 4.26 b
	4	12.08 ± 5.20 c	32.54 ± 4.20 a
	8	9.08 ± 3.17 c	33.63 ± 6.55 a
Wogonin	0	1.60 ± 0.61 d	
	2	0.98 ± 0.12 d	3.06 ± 0.45 c
	4	1.26 ± 0.37 d	4.48 ± 0.43 a
	8	0.73 ± 0.21 d	4.57 ± 1.13 b

Mean values with different letters (a–d) were significantly different (*p* < 0.05, ANOVA, DMRT).

**Table 2:** HPLC analysis of flavones in shoots of *S. baicalensis* plantlets after chilling treatment.

	Duration (Day)	Control	Chilling Treatment
Baicalin	0	0.77 ± 0.05 c	
	2	1.15 ± 0.18 b	1.23 ± 0.08 b
	4	0.83 ± 0.25 c	0.51 ± 0.02 d
	8	0.55 ± 0.37 d	2.01 ± 0.09 a
Baicalein	0	0.04 ± 0.02 c	
	2	0.12 ± 0.05 b	0.12 ± 0.02 b
	4	0.17 ± 0.03 a	0.06 ± 0.02 c
	8	0.04 ± 0.00 c	0.04 ± 0.00 c

Mean values with different letters (a–d) were significantly different (*p* < 0.05, ANOVA, DMRT).

## Data Availability

Not applicable.
